# Comparative genomics reveals conservative evolution of the xylem transcriptome in vascular plants

**DOI:** 10.1186/1471-2148-10-190

**Published:** 2010-06-21

**Authors:** Xinguo Li, Harry X Wu, Simon G Southerton

**Affiliations:** 1 CSIRO Plant Industry, GPO Box 1600, Canberra ACT 2601, Australia

## Abstract

**Background:**

Wood is a valuable natural resource and a major carbon sink. Wood formation is an important developmental process in vascular plants which played a crucial role in plant evolution. Although genes involved in xylem formation have been investigated, the molecular mechanisms of xylem evolution are not well understood. We use comparative genomics to examine evolution of the xylem transcriptome to gain insights into xylem evolution.

**Results:**

The xylem transcriptome is highly conserved in conifers, but considerably divergent in angiosperms. The functional domains of genes in the xylem transcriptome are moderately to highly conserved in vascular plants, suggesting the existence of a common ancestral xylem transcriptome. Compared to the total transcriptome derived from a range of tissues, the xylem transcriptome is relatively conserved in vascular plants. Of the xylem transcriptome, cell wall genes, ancestral xylem genes, known proteins and transcription factors are relatively more conserved in vascular plants. A total of 527 putative xylem orthologs were identified, which are unevenly distributed across the *Arabidopsis *chromosomes with eight hot spots observed. Phylogenetic analysis revealed that evolution of the xylem transcriptome has paralleled plant evolution. We also identified 274 conifer-specific xylem unigenes, all of which are of unknown function. These xylem orthologs and conifer-specific unigenes are likely to have played a crucial role in xylem evolution.

**Conclusions:**

Conifers have highly conserved xylem transcriptomes, while angiosperm xylem transcriptomes are relatively diversified. Vascular plants share a common ancestral xylem transcriptome. The xylem transcriptomes of vascular plants are more conserved than the total transcriptomes. Evolution of the xylem transcriptome has largely followed the trend of plant evolution.

## Background

The evolution of xylem was a critical step that allowed vascular plants to colonize vast areas of the earth's terrestrial surface. Xylem plays an essential role in the transport of water and nutrients and provides mechanical support for vascular plants. Herbaceous vascular plants develop primary xylem, and woody plants also produce secondary xylem (or wood). Evolution from tracheids to vessels reflects a more efficient way for angiosperms to develop secondary xylem [1-3]. Primary xylem consists of cellulose, hemicellulose, pectin and proteins, while secondary xylem contains higher amounts of cellulose and lignin. From a practical point of view, wood represents a renewable natural resource for the timber, fibre and biofuel industries and it is a major carbon sink in natural ecosystems.

In the last few decades the molecular basis of xylem formation and evolution has been investigated. Syringyl (S) lignin biosynthesis was found to be an evolved lignin pathway in angiosperms, representing an addition to the ancient and predominant guaiacyl (G) lignin pathway conserved in land plants [3-6]. However, the S lignin pathway was also recently observed in lycophytes [7], suggesting a complex evolutionary history of lignin biosynthesis in vascular plants. Genes involved in wood formation have been identified in many plant species [8-13], allowing investigation of xylem evolution at the transcriptome level. A core xylem gene set was identified in white spruce among which 31 transcripts are highly conserved in *Arabidopsis *[14]. The expanding genomic resources of model plant species [15-18] are invaluable for exploring many aspects of plant development and evolution, including xylem formation and evolution. Recent studies suggest whole-genome duplication and reorganization [15-17] have been a major driving force in plant evolution.

Comparative genomics is a powerful tool for investigating plant evolution at the whole-genome level [19-27]. Lower collinearity across dicots and monocots [16,21] and lineage-specific genes have been observed using comparative genomics [28,29]. Comparisons between rice and *Arabidopsis *showed that 50% of the rice genome was homologous to *Arabidopsis *[16], while as much as 88% of the poplar genome shares homology with *Arabidopsis *[17]. Comparative genomics of poplar with *Arabidopsis *revealed at least 11,666 protein-coding genes were present in the ancestral eurosid genome [17]. The recently sequenced *Selaginella moellendorffii *[30], moss [31] and *Eucalyptus grandis *[18] genomes provide new opportunities for comparative genomics in vascular plants.

Many commercial crops and forest trees do not have sequenced genomes; thus, comparative genomics in these species must rely on studies of the transcriptome (expressed sequence tags, ESTs). Using a transcriptome approach about half of loblolly pine xylem unigenes did not match *Arabidopsis *unigenes [32], and 39-45% of pine contigs had no hits in the genomes of *Arabidopsis*, rice and poplar [33]. In white spruce and sitka spruce between 30 and 36% of the unigenes lacked homologs in poplar, *Arabidopsis *and rice [12,33]. Similar results were also observed in other comparisons between gymnosperms and angiosperms [19,34]. By contrast, comparison of different gymnosperm species revealed highly conserved transcriptomes. For example, about 84% of the white spruce transcripts had matches in the Pine Gene Index [12], and 60-80% of spruce contigs had homologs in loblolly pine and other gymnosperms [33].

Although comparative genomics has increased our understanding of plant evolution at the genome level, most studies to date have focussed on the entire genome sequence, and/or mixed EST resources developed from a range of tissues. In order to examine xylem evolution in vascular plants we focused our attention on genes expressed specifically in xylem. We selected ten species which represent different categories of vascular plants, including gymnosperms (pine and spruce), angiosperms (woody dicots, herbaceous dicots, and monocots) and lycophytes. The non-vascular plant moss was included as a reference for xylem evolution. Up-to-date public genome sequences and xylem transcriptomes of selected plant species were used for comparative genomic analysis, aiming to explore the evolution of the xylem transcriptome in vascular plants.

## Results

### The xylem transcriptome is highly conserved in conifer species

**Figure 1:**
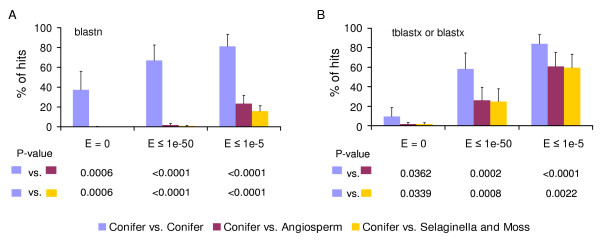
**Comparisons of conifer xylem unigenes with genes in other species**. Xylem unigenes of radiata pine, loblolly pine and white spruce were blasted using blastn (A) and blastx (or tblastx) (B) against xylem unigenes, gene indices, gene models or scaffolds of other plant species. Percentage of hits is presented on the Y-axis at three E-value cut-offs (0, 1e-50 and 1e-5). Eleven databases used for blasts are presented on the X-axis, including pine (PGI) (1) and spruce gene indices (SGI) (2), loblolly pine (3), white spruce (4) and poplar (5) xylem unigenes, *Eucalyptus *scaffolds (7), and gene models of poplar (6), *Arabidopsis *(8), rice (9), *Selaginella *(10) and moss (11), respectively. These databases include four plant groups: conifers (1-4), angiosperms (woody 5-7 and herbaceous 8-9), *Selaginella *(10) and the non-vascular plant, moss (11).

We compared the xylem transcriptomes among different conifer species (radiata pine, loblolly pine and white spruce), and analysed the homologs between conifer xylem transcriptomes and the xylem (or entire) transcriptomes or genomes of angiosperms, lycophytes and moss (Additional file 1A-D and Figure 1A-B). At the nucleotide level 63-66% (E ≤ 10^-50^) and 78-82% (E ≤ 10^-5^) of radiata pine xylem unigenes have homologs in the xylem transcriptomes of loblolly pine and white spruce (Additional file 1A). A higher proportion (74-89% at E ≤ 1e-50, or 93-95% at E ≤ 1e-5) had homologs in the entire transcriptomes of conifers based on gene indices of pine and spruce (Additional file 1A). These results suggest that radiata pine xylem unigenes are highly conserved as full (E ≤ 10^-50^) or partial sequences (E ≤ 10^-5^) in other conifer species. Additional analysis with loblolly pine and white spruce nucleotide sequences (Additional file 1A) revealed similarly high percentages of homologs in other conifer species. Using deduced amino acid sequences more than 58% (E ≤ 10^-50^) or 79% (E ≤ 10^-5^) of the radiata pine xylem transcriptome are conserved as functional genes or domains in the three conifer species. Comparisons of average percentage of homologs among different conifers (Figure 1A-B) further confirmed that the xylem transcriptomes are highly conserved in conifers at both the nucleotide (67% at E ≤ 1e-50, 81% at E ≤ 1e-5) and deduced amino acid sequences (58% at E ≤ 1e-50, 84% at E ≤ 1e-5).

Conifer xylem transcriptomes are significantly distinct from the xylem transcriptome or genomes of angiosperms, lycophytes and moss (Additional file 1A-B). For example, at the nucleotide level only 1-4% (E ≤ 1e-50) and 15-32% (E ≤ 1e-5) of radiata pine xylem unigenes have homologs in angiosperms, *Selaginella *and moss. Furthermore, average percentage of homologs between conifer xylem transcriptomes and angiosperms (xylem transcriptome or entire genome) at the nucleotide level is only 2% (E ≤ 1e-50) or 23% (E ≤ 1e-5) (Figure 1A); while even lower percentages (1% or 16%) are observed between conifers and *Selaginella *& moss. Comparisons between two xylem transcriptomes would be expected to reveal fewer homologs than comparisons between a xylem transcriptome and a total transcriptome or a whole genome. The low percentage of homologs detected is statistically significant (P < 0.001) compared to the high percentage of matches among conifer xylem transcriptomes. Unsurprisingly, the deduced amino acid sequences of conifer xylem unigenes have relatively more homologs in non-coniferous plants (Additional file 1B and Figure 1B). However, they are still significantly (P < 0.001) lower than the homologous xylem unigenes among the three conifers.

We further examined the nucleotide identity of homologous unigenes in the xylem transcriptomes of different conifers as well as homologs between conifer xylem transcriptomes and unigenes of other plant species. The nucleotide identity between radiata pine and loblolly pine is consistently higher (97.5%) at different E-value cut-offs ranging from 0 to 1e-5, than between radiata pine and the two spruce species (91%) (Additional file 1C). By contrast, lower nucleotide identity (81-83%) was observed in the homologs between radiata pine and angiosperms (*Populus*, *Arabidopsis *and rice) or moss. Similar results were also observed in the analyses with loblolly pine (Additional file 1C). The average nucleotide identity of homologous xylem unigenes in different conifers is about 92%, significantly higher (P < 0.01) than the homologs between conifers and angiosperms & moss (about 82%) (Additional file 1D). Thus, comparisons of nucleotide identity provide further evidence of the higher conservation of the xylem transcriptomes in conifers and its divergence in angiosperms and other plants.

### The xylem transcriptome has evolved considerably in angiosperms

**Figure 2:**
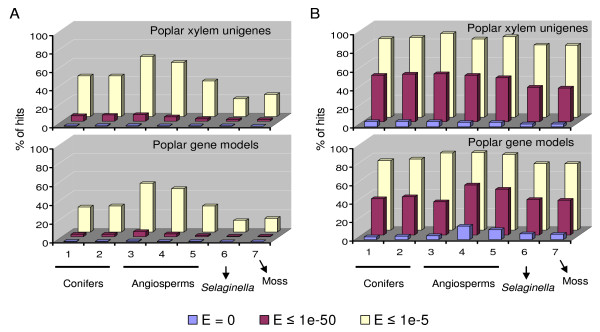
**Comparisons of genes between poplar and other species**. Poplar xylem unigenes and gene models (nucleotide or protein sequences) were blasted with blastn (A) and tblastn (or blastp) (B) against gene indices, gene models or scaffolds of other plant species. Percentage of hits is presented on the Y-axis at three E-value cut-offs (0, 1e-50 and 1e-5). Seven databases used for blasts are presented on the X-axis, including pine (PGI) (1) and spruce gene indices (SGI) (2), *Eucalyptus *scaffolds (3), and gene models of *Arabidopsis *(4), rice (5), *Selaginella *(6) and moss (7), respectively. These databases include four plant groups: conifers (1-2), angiosperms (woody 3 and herbaceous 4-5), *Selaginella *(6) and moss (7).

Poplars are woody angiosperms and are used as a model tree species for wood formation. Surprisingly, at the level of nucleotide sequence less than 5% of poplar xylem unigenes have homologs (E ≤ 1e-50) in the scaffolds or gene models of other woody and herbaceous angiosperms (*Eucalyptus*, *Arabidopsis *and rice) (Figure 2A). Poplar xylem unigenes also have few homologs (E ≤ 1e-50) in the gene indices of pine or spruce and in the gene models of *Selaginella *and moss (Figure 2A). These results suggest that the poplar xylem transcriptome is distinct compared to other vascular and non-vascular plants. Because gene models of a species represent its entire genome which includes the xylem transcriptome, the few homologs shared between the poplar xylem transcriptome and gene models of other angiosperms suggest that the xylem transcriptome has undergone considerable evolution in angiosperms. This contrasts with the highly conserved xylem transcriptomes in gymnosperms observed earlier (Additional file 1A-D and Figure 1A-B). The entire genome of poplar is also unique at the nucleotide level among the species examined in this study (Figure 2A). However, the functional gene sequences of the poplar xylem transcriptome and its entire genome (Figure 2B) are moderately (36-50% at E ≤ 1e-50) conserved, while their functional domains are highly conserved (77-85% at E ≤ 1e-5) in other vascular and non-vascular plants. Clearly, all land plants share an ancestral xylem transcriptome which may have been largely involved in cell wall biosynthesis in ancient non-vascular plants.

### The xylem transcriptome is relatively more conserved in vascular plants

**Figure 3:**
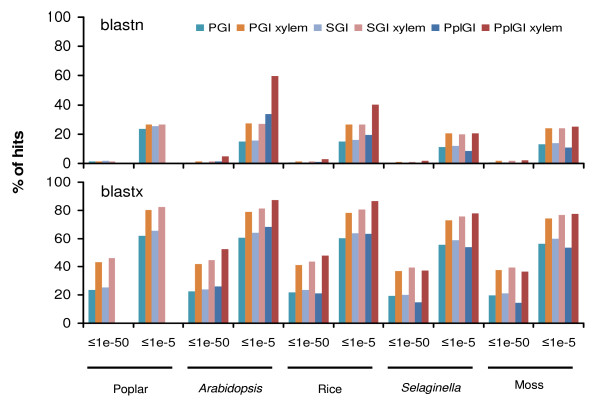
**The xylem transcriptome is relatively more conserved in vascular and non-vascular plants**. Xylem gene indices and total gene indices of pine, spruce and poplar were blasted (blastn and blastx) against the gene models of *Populus*, *Arabidopsis*, rice, *Selaginella *and moss. Percentage of hits is presented on the Y-axis at E-value cut-offs of 1e-50 and 1e-5. Statistical tests for the different number of hits observed with xylem and total gene indices were performed assuming a binomial distribution. All comparisons showed statistical significances (P < 0.001) (data not show for the simplicity of the figure) except for two comparisons between conifer species and poplar (blastn at 1e-50).

We examined the pattern of the xylem transcriptome evolution by comparing it with the total transcriptome derived from a range of tissues. Although the xylem transcriptomes of pine, spruce and poplar have few homologs in the gene models of *Arabidopsis*, rice, *Selaginella *and moss (1.2-5% at E ≤ 1e-50), there are about two to four times more homologs than there are for the total transcriptome (Figure 3). When using a lower stringency E-value (≤ 1e-5), the xylem transcriptomes of pine, spruce and poplar have more homologs (about 20-60%) in the above four model species compared to the total transcriptome (9-34%). Thus, at the nucleotide level the xylem transcriptome is relatively more conserved in vascular and non-vascular plants. Notably both the conifer xylem and total transcriptomes have few homologs in poplar (a woody species) and herbaceous species (*Arabidopsis*, rice, *Selaginella *and moss) (Figure 3), suggesting conifer transcriptomes are distinct from other land plants.

The conservative evolution of the xylem transcriptome was more clearly observed when using deduced amino acid sequences (Figure 3). For example, 37-46% of the conifer xylem transcriptome matched the genomes of angiosperms (*Populus*, *Arabidopsis *and rice), lycophyte and moss; nearly two times more than the matches with the total conifer transcriptome. Similar results were also obtained with the poplar xylem transcriptome in the comparisons with the total transcriptome. Therefore, the functional gene sequences in the xylem transcriptome are significantly more conserved in vascular plants than that in the total transcriptome.

### Different functional gene groups have distinct patterns of evolution

**Figure 4:**
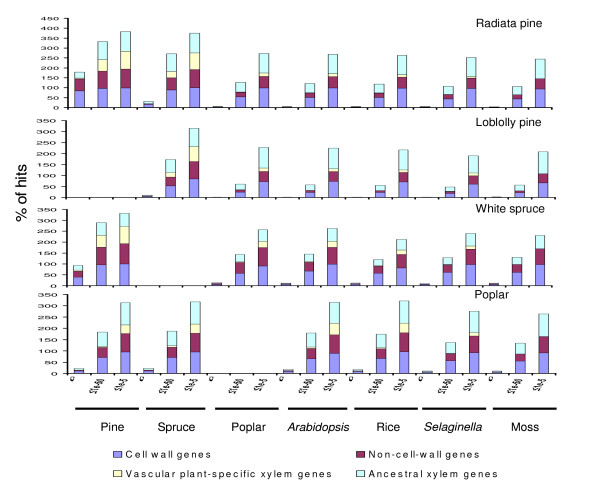
**Cell wall genes and ancestral xylem genes are relatively more conserved in diverse plants**. Putative cell wall, non-cell-wall, vascular plant-specific xylem (VSX) and ancestral xylem genes within xylem unigenes of radiata pine, loblolly pine, white spruce and poplar were blasted with gene indices (tblastx) of pine and spruce, and gene models (blastx) of poplar, *Arabidopsis*, rice, *Selaginella *and moss. Percentage of hits is presented on the Y-axis at three E-value cut-offs (0, 1e-50 and 1e-5). Different numbers of hits observed with cell wall and non-cell-wall genes, as well as with VSX and ancestral xylem genes were statistically tested assuming a binomial distribution. All comparisons showed statistical significances (P < 0.001) (data not show for the simplicity of the figure) except for a few between conifers at 1e-5.

We divided the xylem transcriptome into different functional gene groups for investigating their diverse patterns of evolution. We defined xylem unigenes with matches (blastx, E ≤ 1e-5) in the moss gene models as ancestral xylem genes, and those with no matches as vascular plant-specific xylem (VSX) genes. Among the ancestral xylem genes derived from radiata pine, loblolly pine and white spruce, 96-99%, 78-94% and 48-60% (E ≤ 1e-5), or 41-48%, 20-26% and 28-35% (E ≤ 1e-50), respectively, have homologs (blastx) in the genomes of the three angiosperms and the lycophyte (Figure 4A-C). In contrast, only 8-28% (E ≤ 1e-5) and 0.4-1% (E ≤ 1e-50) of the conifer VSX genes are homologous to the genomes of these model vascular plants (Figure 4A-C). These results suggest that the ancestral xylem genes are moderately to highly conserved in non-coniferous vascular plants, while considerable divergence has occurred in VSX genes among conifers, angiosperms and lycophytes. Similar patterns were observed in the comparisons of VSX and ancestral xylem genes with the poplar xylem transcriptome (Figure 4D).

Cell wall genes were identified according to the CellWallNavigator [35] and MAIZEWALL [36] databases. We compared the level of conservation of cell wall genes to non-cell-wall genes (Figure 4A-D). In the four woody species (radiata pine, loblolly pine, white spruce and poplar) the cell wall genes have 1.5 to two times more homologs than the non-cell-wall genes in the genomes of the four herbaceous model plants (Figure 4A-D). This suggests that cell wall genes have been highly conserved across diverse land plants. Comparisons of transcription factors (TFs) (based on PlantTFDB [37]) with non-TFs and known functional genes with unknowns revealed significantly more conservation of transcription factors and known functional genes among different groups of vascular and non-vascular plants (Additional file 2A-D).

### Identification of putative xylem orthologs in vascular plants

**Figure 5:**
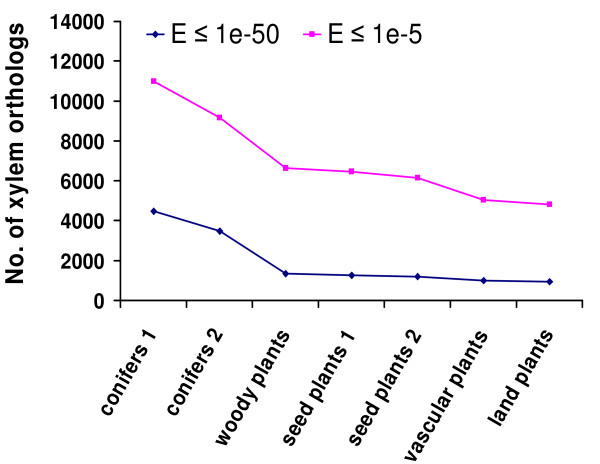
**The number of putative xylem orthologs in different plant groups**. Loblolly pine xylem unigenes (18,320) were blasted using tblastx against the unigenes of white spruce and sitka spruce, and the gene models of poplar, *Arabidopsis*, rice, *Selaginella *and moss. The number of putative xylem orthologs common to different plant groups is presented at two E-value cut-offs (1e-50 and 1e-5). Conifers 1: loblolly pine plus white spruce; Conifers 2: conifers 1 plus sitka spruce; Woody plants: conifers 2 plus poplar; Seed plants 1: woody plants plus *Arabidopsis*; Seed plants 2: seed plants 1 plus rice; Vascular plants: seed plants 2 plus *Selaginella*; Land plants: vascular plants plus moss. The xylem orthologs shared in conifers 2, woody plants, seed plant 1, seed plants 2 and vascular plants represent genes specific to conifers, woody plants, seed plants, lower seed plants (lacking secondary xylem) and vascular plants, respectively. The high proportion of xylem orthologs homologous to moss suggests that the ancestral xylem genome was largely present in non-vascular plants.

Putative xylem orthologs conserved in diverse plant groups were identified using deduced amino acid or protein sequences (Additional file 3), and their numbers are briefly presented at two E-value cut-offs (≤ 1e-50 and ≤ 1e-5) in Figure 5. The xylem orthologs (9,164 at E ≤ 1e-5 or 3,460 at E ≤ 1e-50) shared by loblolly pine, white spruce and sitka spruce represent a common gene set expressed in conifer wood formation. While the xylem orthologs common to the three conifers and poplar (6,641 at E ≤ 1e-5 or 1,339 at E ≤ 1e-50) are putative woody plant xylem orthologs. Surprisingly, the number of putative xylem orthologs in deeper plant groupings is only slightly smaller (Figure 5), suggesting that very limited numbers of xylem orthologs are specific to each plant group. The majority (93-96%) of the vascular plant xylem orthologs have matches in the gene models of a non-vascular plant (moss). This suggests that the xylem transcriptome of vascular plants has largely evolved from a subset of genes found in non-vascular plants. This is likely to be a group of genes predominantly involved in cell wall biosynthesis that have been recruited to synthesize secondary xylem in higher plants.

Of the 1,003 loblolly pine xylem unigenes with homologs (tblastx or blastx, E ≤ 1e-50) in the six vascular plants, 94% and 100% have hits in the UniProt known proteins and TIGR gene indices databases, respectively, and 94% are assigned with GO terms (Additional file 3). Among these unigenes 349 (35%) were identified as cell wall genes, and the largest gene families are *tubulin *(16 unigenes) and *cellulose synthase *(*CesA*) (10). Several primary wall genes are abundant including *pectate lyase *(8), *pectinesterase *(7), *XET *(7), *expansin *(6) and *peroxidase *(5). Lignin biosynthesis-related genes are well represented in the cell wall gene lists, including *SAMS *(11), *laccase *(9), *methionine synthase *(*cobalamin-independent*) (6), *4CL *(3), *COMT *(2), *CAD *(2) and *CCoAMT *(3). In addition, 49 transcription factors were found, including NAC, PHD, HB, MYB, LIM, CCCH, etc. The large number of transcription factors suggests that wood formation involves considerable transcriptional regulation. Interestingly, *aquaporins *(16 unigenes) is one of the largest gene families in the 1,003 unigenes, reflecting their central importance in water transport in xylem.

The 1,003 loblolly pine xylem unigenes have between 749 and 894 close homologs (blastx or tblastx, E ≤ 1e-50) in white spruce, sitka spruce, poplar, *Arabidopsis*, rice and *Selaginella*, with 527 xylem orthologs common to the above 6 species. These common xylem orthologs matched (blastx, E ≤ 1e-50) 501 unique gene models in moss, indicating that at least 501 ancestral xylem orthologs are shared in land plants. We also identified all loci in *Arabidopsis *and rice with homology to the 1,003 loblolly pine xylem unigenes. All possible homologs in *Arabidopsis *are represented by 3,115 (E ≤ 1e-50) or 6,563 unique loci (E ≤ 1e-5), and in rice by 3,057 or 8,458 unique loci. Based on the numbers of close homologs in *Arabidopsis *(785) and rice (776), an average of 4-8 and 4-11 paralogs may have arisen from each xylem ortholog in these two species, respectively.

**Figure 6:**
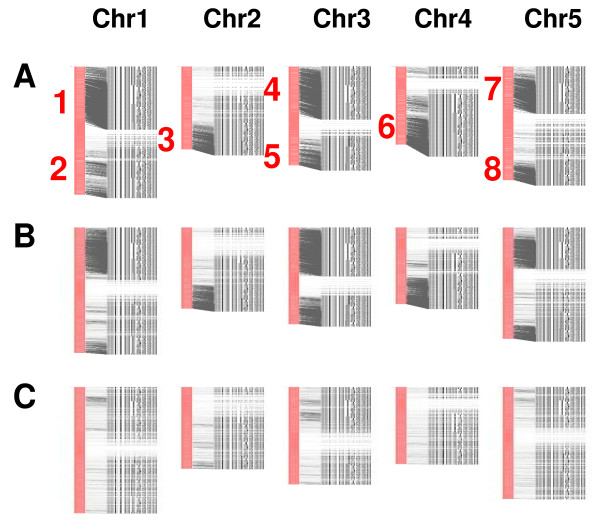
**Hot spots of putative xylem orthologs on the chromosomes of *Arabidopsis***. The 1,003 loblolly pine xylem unigenes highly conserved in other vascular plants (xylem orthologs) have strong homology (1e-50) with 3,115 loci and moderate homology (1e-5) with 6,563 loci in the *Arabidopsis *genome. Positioning of these homologs on the five chromosomes of *Arabidopsis *revealed eight hot spots of putative *Arabidopsis *xylem orthologs. A: 3,115 highly homologous loci; B: 6,563 moderately homologous loci; C: 6,000 randomly selected loci.

The xylem orthologs and paralogs are relatively evenly distributed across the chromosomes of *Arabidopsis *and poplar, but may be unevenly distributed among rice chromosomes (Additional file 4). However, eight hot spots were detected within the five *Arabidopsis *chromosomes (Figure 6A, B), which contrasts with the even distribution of all *Arabidopsis *genes across each chromosome [38] (Figure 6C). These hot spots may be a result of gene and chromosome duplication in *Arabidopsis *[15,39,40]. Within the chromosomes of rice and poplar the xylem orthologs have a relatively even distribution (Additional file 5A-C and 6A-C). Different distribution patterns of the xylem orthologs among the *Arabidopsis*, rice and poplar genomes may reflect their diverse history of gene duplication and segmental chromosomal rearrangements.

### Molecular evolution of xylem orthologs in vascular plants

**Figure 7:**
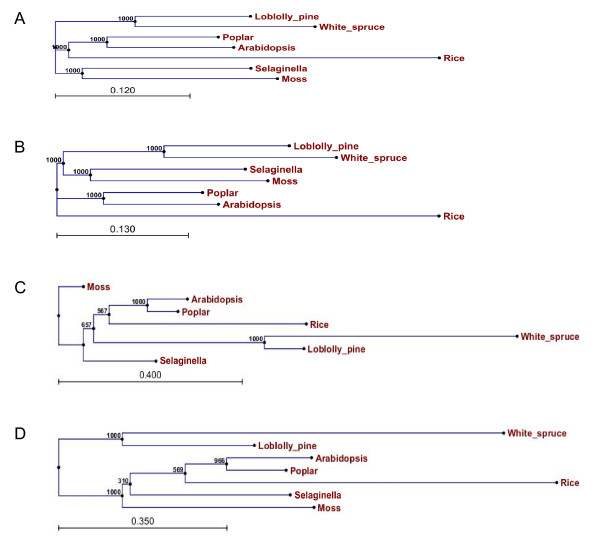
**Phylogenetic trees for ancestral xylem orthologs in land plants**. Deduced amino acid or protein sequences of 501 ancestral xylem orthologs from 7 species were combined to build consensus trees using three alignment methods, including Kalign (A), ClustalX2 and Mafft (B) with a bootstrap of 1,000. Individual trees were also generated for each gene using CLC Genomic Workbench 3 (CLC bio, Denmark). Genes with phylogenetic trees paralleled the trend of plant evolution (235, 47%) were present in an example (C) while genes that diverged from plant evolution (266, 53%) had diverse patterns of molecular evolution. A representative phylogenetic tree of genes (158, 32%) with conserved branches between angiosperms and ancient plant species but divergent branches to gymnosperms is showed in example (D).

We used the 501 ancestral xylem orthologs for phylogenetic analysis because they largely (95%) represent the 527 unique xylem orthologs and it allowed us to include the non-vascular plant moss as a reference. Two slightly different trees (Figure 7A-B) were constructed using three alignment methods, two of which generated the same tree configuration (Figure 7B). These trees clearly separate angiosperms and gymnosperms from lycophytes and moss. Based on tree B moss and *Selaginella *emerged at the earliest time point followed by the two conifers (loblolly pine and white spruce) and then by the two dicots (poplar and *Arabidopsis*). The monocot xylem transcriptome appears to be the most evolutionarily distinct. In summary, the phylogenetic analysis suggests that molecular evolution of xylem orthologs largely parallels the trend of plant evolution.

From individual phylogenetic trees of 501 ancestral xylem orthologs, 235 (46.9%) genes showed an evolutionary pattern that generally followed the trend of plant evolution (Figure 7C). These genes are likely more sensitive to evolutionary forces. Molecular evolution of other xylem orthologs (266, 53.1%) did not closely resemble the general pattern of plant evolution. A large proportion of orthologs in this category (158, 31.5%) had trees with similar patterns between angiosperms (poplar, *Arabidopsis *and rice) and ancient plant species (moss and/or *Selaginella*), but quite distinct branches for the gymnosperms (pine and spruce) (Figure 7D). These genes have possibly undergone slower evolution, although considerable evolution appears to have occurred within the gymnosperms. This type of genes may have unique and important roles in softwood formation. Other orthologs (108, 21.6%) exhibit complex evolutionary patterns which are poorly correlated with plant evolution, suggesting that genetic shift might be a major driven force for their evolution.

Using GO terms at various levels we compared gene functions between xylem orthologs which paralleled and diverged from plant evolution. Genes involved in cellular components and biological process are significantly (P-value < 0.05) more abundant in the divergent orthologs (Additional file 7). Further analyses with GO terms at lower levels (Additional file 7) suggest that genes involved in ribosome, transporter activity and translation tend to parallel plant evolution; while genes with functions in cell parts, binding and metabolic process have distinct patterns of evolution. Among cell wall genes *4CL-like*, *XET *and *beta-galactosidase *have a molecular evolutionary pattern paralleling plant evolution. However, many other cell wall genes have not tightly followed plant evolution, such as *CCoAMT*, *pectate lyase*, *pectinesterase*, *sucrose synthase*, *actin*, *alpha tubulin*, *COMT*, *CesA*, *FLA*, *laccase *and *peroxidase*.

### Identification of putative conifer-specific xylem genes

A total of 274 loblolly pine xylem unigenes which matched unigenes of white spruce and sitka spruce (tblastx, E ≤ 1e-50), but have no homologs in the gene models of poplar, *Arabidopsis*, rice, *Selaginella *and moss, as well as no hits in any other angiosperms in the UniProt known protein database (blastx, E > 1e-5), were identified as putative conifer-specific xylem unigenes (Additional file 8). All conifer-specific xylem unigenes are unknown protein sequences based on the UniProt database, and only 16% were assigned with GO terms in the TIGR gene index database. This contrasts with 94% of the xylem orthologs annotated with GO terms. The poor annotation of the conifer-specific xylem genes is likely due to the intense functional characterisation of genes that has taken place in angiosperms.

Of the conifer-specific xylem unigenes several relatively abundant transcripts have low homology to arabinogalactan-like proteins (AGPs), glycine/proline-rich proteins (GPRP), metallothionein-like class II proteins, neurofilament triplet H proteins, zinc finger proteins, cytochrome c1, etc (Additional file 8). Genes with low similarity to other cell wall proteins (such as alpha tubulin, cellulose synthase like and extensin-like proteins) are present. The conifer-specific xylem unigenes also include genes similar to transcription factors, i.e. Aux/IAA, NAC, CCAAT-binding, WRKY, R2R3-MYB, BHLH and CCAAT-binding (Additional file 8). These conifer-specific xylem unigenes may share the functions of related homologues, but some may have unique roles in gymnosperm wood formation.

## Discussion

### Gymnosperms and angiosperms have distinct patterns of xylem transcriptome evolution

Our data revealed that the xylem transcriptome is highly conserved in conifers but has undergone considerably more diversification in angiosperms. This suggests the xylem transcriptome has a distinct pattern of evolution in angiosperms compared to gymnosperms. Pine and spruce diverged 120-140 Ma [41], slightly earlier than the radiation of angiosperms (100-120 Ma) [16,17]. The rapid evolution of the angiosperm xylem transcriptome suggests greater sensitivity to evolutionary forces in flowering plants. Our results also showed that poplar has a relatively distinct genome and xylem transcriptome, thus poplar is unlikely to be a useful model for investigating wood formation and other developmental processes in gymnosperms. Mechanisms underlying the diverse patterns of xylem transcriptome evolution in angiosperms and gymnosperms are not well understood. Gene duplications and segmental chromosome rearrangements may be the major driving forces for this diversification; however, the role of genome size or gene number remains unclear.

### The common ancestor of the xylem transcriptome in vascular plants

Our data demonstrated that the nucleotide and protein sequences of the xylem transcriptome are highly diversified among different categories of vascular plants, but that their functional domains are moderately to highly conserved in vascular plants. This is consistent with the diversification of all vascular plants from a common ancestor which is thought to have lived about 400 Ma [42]. Some of these conserved patterns are also consistent with comparisons using the total transcriptome in previous studies [32,43]. The conserved functional domains of the xylem transcriptome suggest the existence of a shared ancestral xylem transcriptome of vascular plants.

The common ancestral xylem transcriptome should be present in the earliest ancient vascular plants, which produced hilate/trilete spores during the Late Ordovician [44]. The lycophyte *Selaginella *is among the closest living relatives of these plants and its xylem transcriptome is likely to resemble most closely the ancestral xylem transcriptome. The 5,047 (27%) xylem unigenes of loblolly pine with homologs (E ≤ 1e-5) in the six vascular plants including *Selaginella *could largely represent the ancestral xylem transcriptome. Because the vast majority (95%) of the ancestral xylem transcriptome has homologs (E ≤ 1e-5) in the moss genome, the xylem transcriptome is likely to have evolved largely from the cell wall transcriptome of non-vascular plants, which can be traced back as far as 450 Ma [31].

### Conservative evolution of the xylem transcriptome in vascular plants

Xylem functions in the transport of water and solutes throughout the plant and together with the phloem makes up the vascular transport tissues of plants. Our data suggests that the xylem transcriptome is relatively more conserved than the total transcriptome in vascular plants. Several functional gene groups within the xylem transcriptome are significantly more conserved among vascular plants, including cell wall genes, ancestral xylem genes, transcription factors and known protein genes. This implies that xylem has evolved more slowly compared to other organs of vascular plants such as leaves, flowers and seeds. On the other hand, the unknown function and VSX genes have been more rapidly evolving in vascular plants. The rapid evolution of VSX genes contrasts with the generally conservative evolution of the total xylem transcriptome, suggesting VSX genes are particularly sensitive to evolutionary forces. These genes will be useful targets for functional characterisation in order to understand xylem formation and evolution in vascular plants.

Our data showed that the loblolly pine xylem transcriptome has significantly more homologs in spruce (white spruce and sitka spruce) than in poplar and *Eucalyptus*, thus it is possible to identify genes specific to gymnosperm wood formation (softwoods). Interestingly, the number of homologs of the loblolly pine xylem transcriptome in woody angiosperms (poplar and *Eucalyptus*) is similar to that in herbaceous angiosperms (*Arabidopsis *and rice), suggesting that evolution in a small number of xylem genes in angiosperms gave rise to woody plants. Furthermore, the number of homologs of the conifer xylem transcriptome in angiosperms is only slightly more than the number in lycophytes and moss. The limited number of xylem genes specific to woody plants suggests that differential transcriptional regulation may have played an important role in xylem evolution; in a similar way that transcriptional regulation gives rise to plant diversity [20].

### Comparison of gene expression between softwoods and hardwoods

A previous study in *Cryptomeria japonica *identified 56 putative conifer-specific transcripts, including three specific to reproductive organs and one (unknown) specific to woody tissues [45]. Here we identified 274 conifer-specific xylem unigenes, all of which are of unknown function. Transcripts with low homology to some cell wall genes (i.e. *tubulin*, *CesA *and *AGP*) are abundant or present in the conifer-specific xylem unigenes (Additional file 8). These cell wall gene families may include some unique members which are specific to conifer wood formation. The identified conifer-specific xylem genes could provide clues to the molecular processes that give rise to the distinct cell wall structures and chemical properties of softwoods compared to hardwoods. Genes related to lignin biosynthesis are not present in the conifer-specific xylem unigenes, which is consistent with the earlier conclusion that the conifer lignin pathway is conserved in other vascular plants [3].

Transcripts similar to *arabinogalactan protein *(*AGP*) genes are the most abundant genes in the conifer-specific xylem unigenes. In loblolly pine six *AGPs *including *PtAGP4 *were identified in xylem tissues [46] and 11 *AGPs *were identified in radiata pine. Radiata pine *PrAGP4 *is highly abundant in earlywood [13] and preferentially transcribed in earlywood at different tree ages across a rotation period [47]. The conifer-specific xylem *AGPs *and *AGP-like *genes had no homologs with any of the 47 *AGPs *of *Arabidopsis *[48-50]. AGPs are a large class of hydroxyproline-rich glycoproteins. Most AGPs are anchored to the plasma membrane [48] and released into the cell wall after cleavage of the GPI anchor [51]. AGPs may act as cell wall plasticizers, enlarging the pectin matrix, and allowing wall extension and cell expansion [52]. Radiata pine AGPs are located in the compound middle lamella of newly developed tracheids [53]. Expression of AGPs containing FLA domains has recently been found to influence the mechanical strength of stems of the herbaceous (*Arabidopsis*) and woody (*Eucalyptus*) angiosperms [54]. However, fasciclin-like domains (FLAs) were not found in the *AGP *genes specific to conifer wood formation, suggesting different AGPs may have distinct roles in softwood and hardwood formation.

We identified 527 xylem orthologs in vascular plants, including many primary and secondary cell wall genes as well as transcription factors. Conservation of cell wall genes suggests that cell wall biosynthesis is a central event in xylem formation in vascular plants. These xylem orthologs maintain the stability of the basic xylem machinery in vascular plants and may regulate development of xylem structures and properties shared by different vascular plants, such as the common features of softwoods and hardwoods. COMT was previously thought to be one of three enzymes (F5H/Cald5 H, COMT and SAD) specifically involved in S lignin synthesis of angiosperms [3]. The occurrence of *COMT *genes in the xylem orthologs of vascular plants suggests their involvement in diverse functions other than S lignin synthesis.

## Conclusions

The xylem transcriptome is highly conserved in conifer species, but the nucleotide sequences of the xylem transcriptome are significantly distinct among angiosperms, thus considerable evolution of the xylem transcriptome has occurred in angiosperms. The functional domains of the xylem transcriptome are moderately to highly conserved among vascular and non-vascular plants. This suggests that vascular plants share an ancestral xylem transcriptome which is likely to have evolved predominantly from the cell wall transcriptome of non-vascular plants. In comparison to the total transcriptome from a wide range of tissues, the xylem transcriptome has evolved conservatively in vascular plants. Several functional gene groups within the xylem transcriptome, including cell wall genes, ancestral xylem genes, transcription factors and known function genes, are relatively more conserved in vascular plants. A total of 527 xylem orthologs of vascular plants and 274 conifer-specific xylem genes were identified in this study. These genes provide good candidates for molecular investigations of xylem formation and evolution in softwoods (conifers) and hardwoods (woody angiosperms). The uneven distribution of xylem orthologs within *Arabidopsis *chromosomes suggests genome rearrangements have played an important role in xylem evolution. Phylogenetic analysis of the 501 ancestral xylem orthologs suggests that molecular evolution of the xylem transcriptome has largely paralleled the trend of plant evolution despite several gene classes did not tightly track it.

## Methods

### Plant species

We selected ten species to represent three major classes of vascular plants (gymnosperms, angiosperms and lycophytes). Four conifer species: *Pinus radiata *(radiata pine), *Pinus taeda *(loblolly pine), *Picea glauca *(white spruce) and *Picea sitchensis *(sitka spruce), represent gymnosperms. Four species of dicots including *Populus tremula × Populus tremuloides *(hybrid aspen), *Populus trichocarpa*, *Eucalyptus grandis *and *Arabidopsis thaliana*, and one monocot (*Oryza sativa*, rice) represent angiosperms. The recently sequenced *Selaginella moellendorffii *[30] represents lycophytes. The non-vascular plant *Physcomitrella patens *(moss) was included as a reference. The seven woody species (four conifer and three woody dicot species) both undergo primary and secondary xylem formation. *Arabidopsis *also develops secondary xylem and has been used as a model system for secondary xylem development [10,11,55-58]. The monocot rice and the lycophyte *S. moellendorffii *only produce primary xylem, while the non-vascular plant moss has no xylem at all.

### Public genomic resources

We previously developed a radiata pine xylem transcriptome resource with 3,304 xylem unigenes [13]. Unigenes of loblolly pine, white spruce, sitka spruce, hybrid aspen, *P. trichocarpa*, *Arabidopsis*, rice and moss were retrieved from the NCBI UniGene database [59] (Additional file 9). The gene indices of pine, spruce and poplar (Additional file 9) were collected from the TIGR gene indices database [60]. These gene indices represent the total transcriptome from a wide range of tissues (such as stem, shoots, xylem, leaves, flowers, bark, roots and seeds, etc). A sub-set of xylem gene indices of pine, spruce and poplar was separately retrieved from pure xylem libraries. These included genes expressed in pure xylem tissues, while genes expressed in mixed tissues of xylem and phloem (such as shoots, cambial regions, etc) were not considered as they were likely to contain phloem-specific transcripts. After removing redundant sequences a total of 14,527, 15,262 and 9,109 xylem gene indices of pine, spruce and poplar were identified. To minimize the bias in comparative genomics, pure xylem gene indices were reassembled using the same method and parameters as used in the EST assembly of radiata pine [13]. A total of 18,320, 12,489 and 7,991 pure xylem unigenes were finally obtained for pine, spruce and poplar, respectively.

The public transcriptome resources (unigenes and gene indices) are unlikely to include all transcripts expressed in xylem formation due to sampling limitations. Therefore, selected fully sequenced plant species (*P. trichocarpa*, *E. grandis*, *Arabidopsis*, rice, *S. moellendorffii *and *P. patens*) were added for comparative genomics in this study (Additional file 9). Gene models of *P. trichocarpa*, *S. moellendorffii *and *P. patens *(moss) were downloaded from the JGI website [61]. *E. grandis *scaffolds were downloaded from EucalyptusDB [18]. *A. thaliana *gene models were downloaded from the TAIR website [62] and *O. sativa *gene models were downloaded from the MSU website [63]. Detailed information on the public genomic resources used in this study is listed in Additional file 9.

### Comparative genomic analysis

Comparisons of sequences were performed locally using the BlastStation2 software (TM software, Inc., CA) with default parameters, including various blast programs (blastn, tblastx, blastx, blastp and tblastn). Expected-value (E-value or E), sequence identity and bit scores were collected for evaluating the similarity between two sequences. Percentage of hits, average sequence identity and average bit score at different E-value cut-offs were calculated for the comparisons of transcriptomes or genomes between different plant species.

The E-value cut-offs for inferring sequence similarity vary from 1e-3 [17,24] to 1e-50 [64,65], but 1e-10 to 1e-30 have been widely used [19,32,43,66-69]. To ensure high confidence as previously suggested [64] and to increase the likelihood of inferring biological significance, we used the following thresholds of E-values: (a) E = 0, the two sequences are identical and considered to derive from the same gene; (b) 1e-50 ≤ E < 0, the two sequences are highly similar and likely to be from the same gene family; (c) 1e-5 ≤ E < 1e-50, the two sequences are similar in one or more regions along the whole sequence and likely to contain the same functional domain(s) or motif(s); (d) E < 1e-5, the two sequences are likely to be unrelated genes. As a general rule, E ≤ 1e-50 was interpreted in this study to indicate whole sequence similarity of two given sequences (gene conservation or apparent homologs); and 1e-5 to 1e-50 was interpreted to indicate partial sequence similarity (domain or motif conservation). Possible influences of different blast programs and the size of databases were not considered in setting the above E-value thresholds.

### Phylogenetic analysis

Phylogenetic analysis of the ancestral xylem orthologs was carried out using the predicted amino acid sequences of loblolly pine and white spruce, and protein sequences from the five model species (poplar, *Arabidopsis*, rice, *Selaginella *and moss). For each species all these sequences were combined into one sequence. The sequences from the above 7 species were aligned using three methods: ClustalX2, Kalign and Mafft [70] with default settings. Phylogenetic trees were created using the neighbour-joining algorithm with a bootstrap of 1,000. Sequences of individual genes were also used to build phylogenetic trees using CLC Genomics Workbench 3 (CLC bio, Denmark). All individual trees were visually compared with the trees from the combined sequence data.

## Abbreviations

Mb: million base pairs; Ma: million years ago; EST: expressed sequence tag; E: E-value or expected-value; GO: gene ontology; PGI: pine gene index; SGI: spruce gene index; PplGI: *Populus *gene index; PGI xylem: gene index from pure xylem tissues of pines; SGI xylem: gene index from pure xylem tissues of spruces; PplGI xylem: gene index from pure xylem tissues of *Populus*; VSX genes: vascular plant-specific xylem genes; TF: transcription factor; CesA: cellulose synthase; AGP: arabinogalactan protein; COMT: caffeic acid 3-O-methyltransferase; FLA: fasciclin-like arabinogalactan protein; XET: xyloglucan endotransglucosylase/hydrolase; CCoAMT: caffeoyl-CoA O-methyltransferase; 4CL: 4-coumarate-CoA ligase; SAMS: S-adenosyl-L-methionine synthetase; CAD: cinnamyl alcohol dehydrogenase.

## Authors' contributions

XL contributed the project idea, collected relevant sequence data, conducted the comparative analysis and prepared the manuscript. SS and HW proposed and guided the research project. All the authors have approved the final manuscript.

## Supplementary Material

Additional file 1 **Comparisons of xylem unigenes in conifers and genes in other species**. The xylem transcriptomes of radiata pine, loblolly pine and white spruce were blasted using blastn (A) and blastx (or tblastx) (B) against xylem unigenes, gene indices, gene models or scaffolds of other plant species. Eleven databases were used for blasts and presented on the X-axis, including pine (PGI) (1) and spruce gene indices (SGI) (2), xylem unigenes of loblolly pine (3), white spruce (4) and poplar (5), poplar gene models (6), *Eucalyptus *scaffolds (7), and gene models of *Arabidopsis *(8), rice (9), *Selaginella *(10) and moss (11). These databases include four plant groups: conifers (1-4), angiosperms (woody 5-7 and herbaceous 8-9), *Selaginella *(10) and the non-vascular plant, moss (11). Percentage of hits is presented on the Y-axis at three E-value cut-offs (0, 1e-50 and 1e-5). In addition, the xylem transcriptomes of radiata pine and loblolly pine were blasted (blastn) against unigenes (the entire transcriptome) of other plant species, including loblolly pine (1), white spruce (2), sitka spruce (3), hybrid aspen (4), *P. trichocarpa *(5), *Arabidopsis *(6), rice (7) and moss (8). These databases were presented on the X-axis including three plant groups: conifers (1-3), angiosperms (woody 4-5 and herbaceous 6-7) and moss (8). Homologous unigenes at four E-value cut-offs (0, 1e-100, 1e-50 and 1e-5) were collected. Nucleotide identities (%) in each comparison (C), average nucleotide identities (%) within a plant group and their variations (D) were presented on the Y-axis. Error bar in figure D indicates standard deviation of average nucleotide identities, and statistical test was shown using P-values. Click here for file

Additional file 2 **Known protein genes and transcription factors in the xylem transcriptome are relatively more conserved in diverse plants**. Known protein genes, unknowns, transcription factors (TFs) and non-TFs of radiata pine, loblolly pine, white spruce and poplar were blasted against gene indices (tblastx) of pine and spruce, and gene models (blastx) of poplar, *Arabidopsis*, rice, *Selaginella *and moss. Percentage of hits is presented on the Y-axis at three E-value cut-offs (0, 1e-50 and 1e-5). Different numbers of hits observed with known protein genes and unknowns, as well as with TFs and non-TFs were statistically tested assuming a binomial distribution. Almost all comparisons are statistically significant (P < 0.001) except for a few comparisons between conifers at 1e-5. Click here for file

Additional file 3 **The 527 unique xylem orthologs of vascular plants**. A total of 1,003 loblolly pine xylem unigenes have homologs (tblastx or blastx, E ≤ 1e-50) in the unigenes or gene models of white spruce, sitka spruce, poplar, Arabidopsis, rice and Selaginella. These unigenes represent possibly 527 unique xylem orthologs in vascular plants. The 527 xylem orthologs matched 501 unique gene models of the non-vascular plant, moss. Click here for file

Additional file 4 **Distribution of xylem orthologs on each chromosome of *Arabidopsis*, rice and poplar**. The proportion of genes homologous (E ≤ 1e-50 or 1e-5) to the 527 xylem orthologs in each chromosome of *Arabidopsis*, rice and poplar was compared to the proportion of all genes in each chromosome. Their differences were statistically tested and those showing significance were indicated in the figure using P-values. Click here for file

Additional file 5 **Location of xylem orthologs on rice chromosomes**. Location of genes homologous to the 527 xylem orthologs in each chromosome of rice. A: 3,057 stronger homologs based on E ≤ 1e-50; B: 8,458 moderate homologs based on E ≤ 1e-5; C: 3,000 randomly selected loci. Click here for file

Additional file 6 **Location of xylem orthologs on poplar chromosomes**. Location of genes homologous to the 527 xylem orthologs in each chromosome of poplar. A: 3,523 stronger homologs based on E ≤ 1e-50; B: 7,436 moderate homologs based on E ≤ 1e-5; C: 3,200 randomly selected loci. Click here for file

Additional file 7 **Comparisons of gene ontology (GO) terms of ancestral xylem orthologs with evolutionary patterns that parallel or diverge from the trend of plant evolution**. Among 501 ancestral xylem orthologs 235 genes have a molecular evolution pattern similar to that of plant evolution, while 266 genes diverge from plant evolution. Functions of genes in these two groups were compared at different levels of GO terms and their differences were statistically tested using P-values. Click here for file

Additional file 8 **The 274 putative conifer-specific xylem unigenes**. A total of 274 loblolly pine xylem unigenes which matched unigenes of white spruce and sitka spruce (tblastx, E ≤ 10^-50^), but have no homologs in the gene models of poplar, *Arabidopsis*, rice, *Selaginella *and moss as well as other angiosperm species in the UniProt known protein database (blastx, E > 10^-5^), were identified as putative conifer-specific xylem unigenes. Click here for file

Additional file 9 **Public plant genomic resources used in this study**. A total of 11 plant species representing different plant groups were selected for this study. Up-to-date public genomic databases were downloaded for comparative genomics analyses. The version and other basic information of these databases are indicated in the table. Click here for file
