# Supplementary figures and images for: Comparative genomics reveals conservative evolution of the xylem transcriptome in vascular plants
Source: BMC Evol Biol. 2010 Jun 21;10:190. doi: 10.1186/1471-2148-10-190 (PMC2907377; doi:10.1186/1471-2148-10-190)

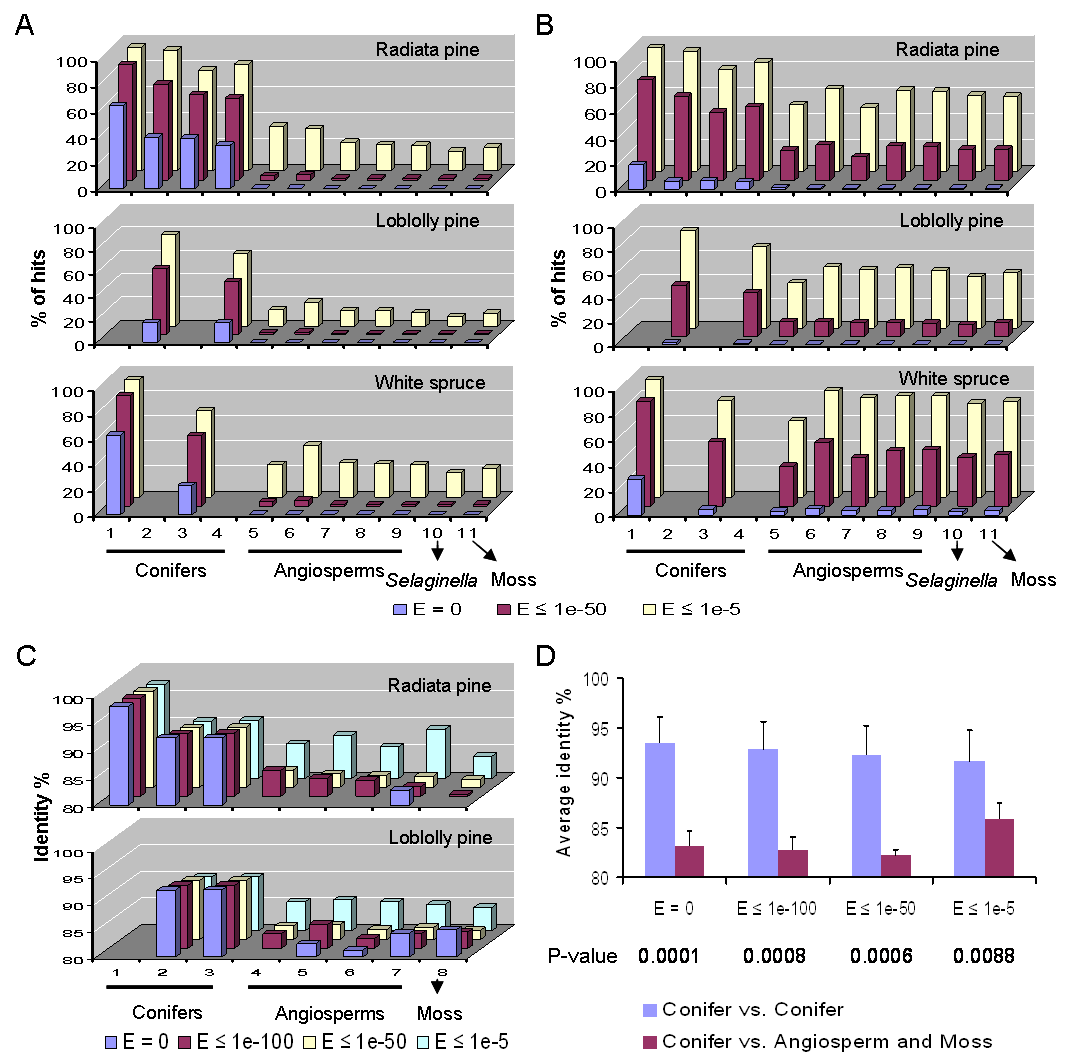

Supplement: Additional file 1 — Comparisons of xylem unigenes in conifers and genes in other species. The xylem transcriptomes of radiata pine, loblolly pine and white spruce were blasted using blastn (A) and blastx (or tblastx) (B) against xylem unigenes, gene indices, gene models or scaffolds of other plant species. Eleven databases were used for blasts and presented on the X-axis, including pine (PGI) (1) and spruce gene indices (SGI) (2), xylem unigenes of loblolly pine (3), white spruce (4) and poplar (5), poplar gene models (6), Eucalyptus scaffolds (7), and gene models of Arabidopsis (8), rice (9), Selaginella (10) and moss (11). These databases include four plant groups: conifers (1-4), angiosperms (woody 5-7 and herbaceous 8-9), Selaginella (10) and the non-vascular plant, moss (11). Percentage of hits is presented on the Y-axis at three E-value cut-offs (0, 1e-50 and 1e-5). In addition, the xylem transcriptomes of radiata pine and loblolly pine were blasted (blastn) against unigenes (the entire transcriptome) of other plant species, including loblolly pine (1), white spruce (2), sitka spruce (3), hybrid aspen (4), P. trichocarpa (5), Arabidopsis (6), rice (7) and moss (8). These databases were presented on the X-axis including three plant groups: conifers (1-3), angiosperms (woody 4-5 and herbaceous 6-7) and moss (8). Homologous unigenes at four E-value cut-offs (0, 1e-100, 1e-50 and 1e-5) were collected. Nucleotide identities (%) in each comparison (C), average nucleotide identities (%) within a plant group and their variations (D) were presented on the Y-axis. Error bar in figure D indicates standard deviation of average nucleotide identities, and statistical test was shown using P-values. [file 1471-2148-10-190-S1.TIFF]

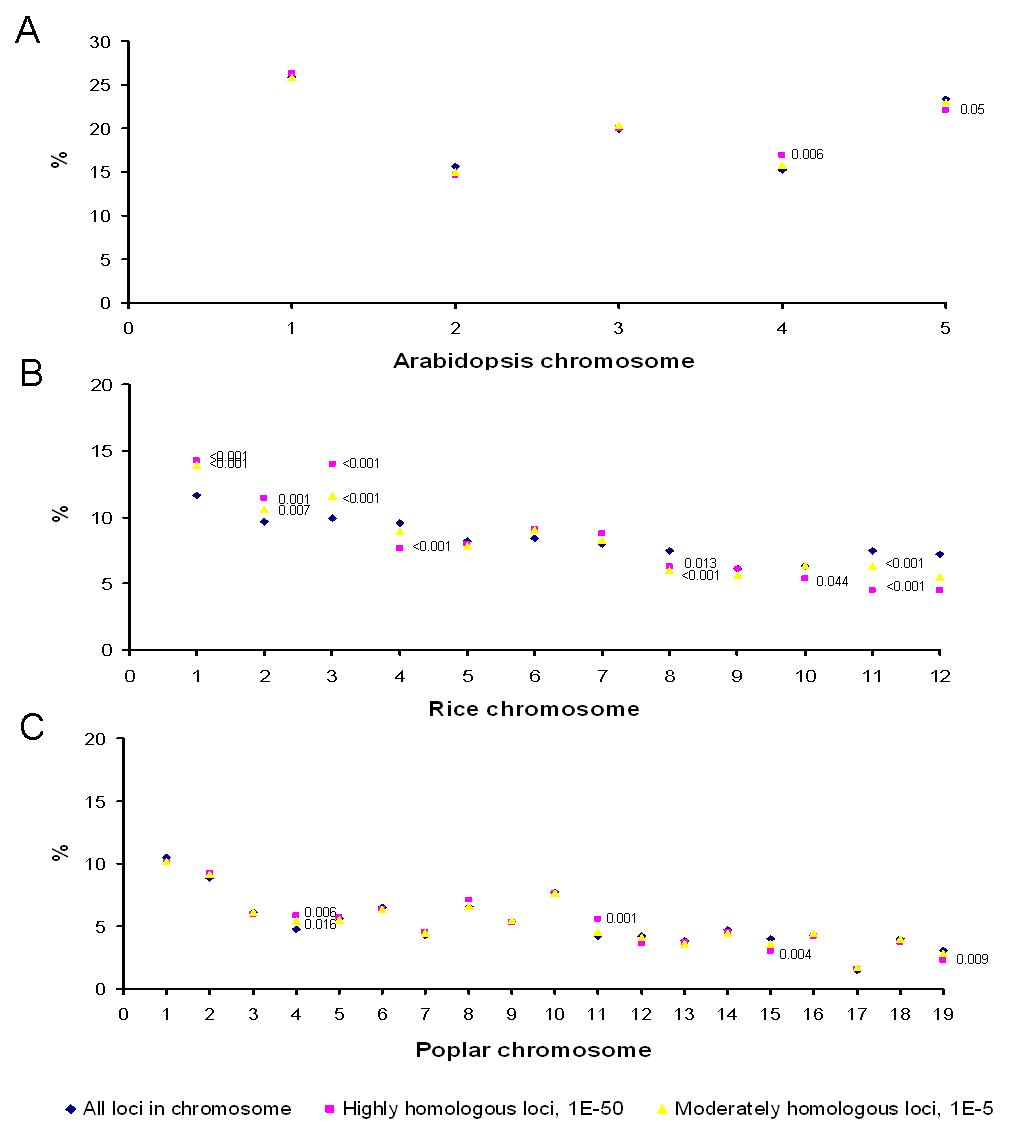

Supplement: Additional file 4 — Distribution of xylem orthologs on each chromosome of Arabidopsis, rice and poplar. The proportion of genes homologous (E ≤ 1e-50 or 1e-5) to the 527 xylem orthologs in each chromosome of Arabidopsis, rice and poplar was compared to the proportion of all genes in each chromosome. Their differences were statistically tested and those showing significance were indicated in the figure using P-values. [file 1471-2148-10-190-S4.TIFF]

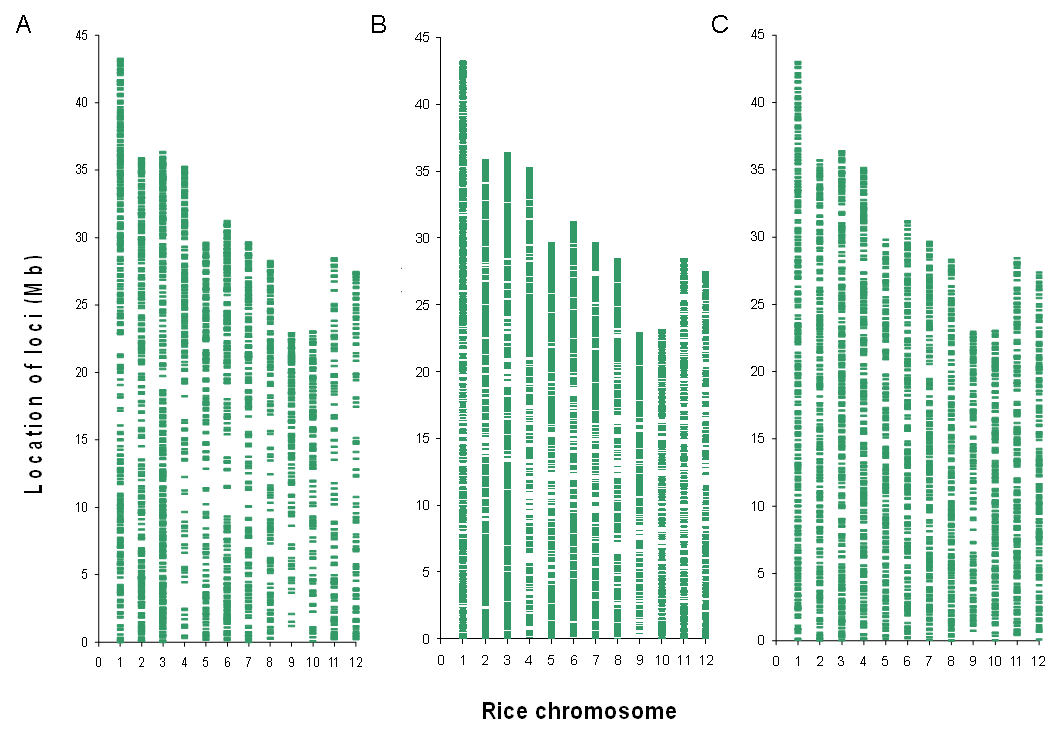

Supplement: Additional file 5 — Location of xylem orthologs on rice chromosomes. Location of genes homologous to the 527 xylem orthologs in each chromosome of rice. A: 3,057 stronger homologs based on E ≤ 1e-50; B: 8,458 moderate homologs based on E ≤ 1e-5; C: 3,000 randomly selected loci. [file 1471-2148-10-190-S5.TIFF]

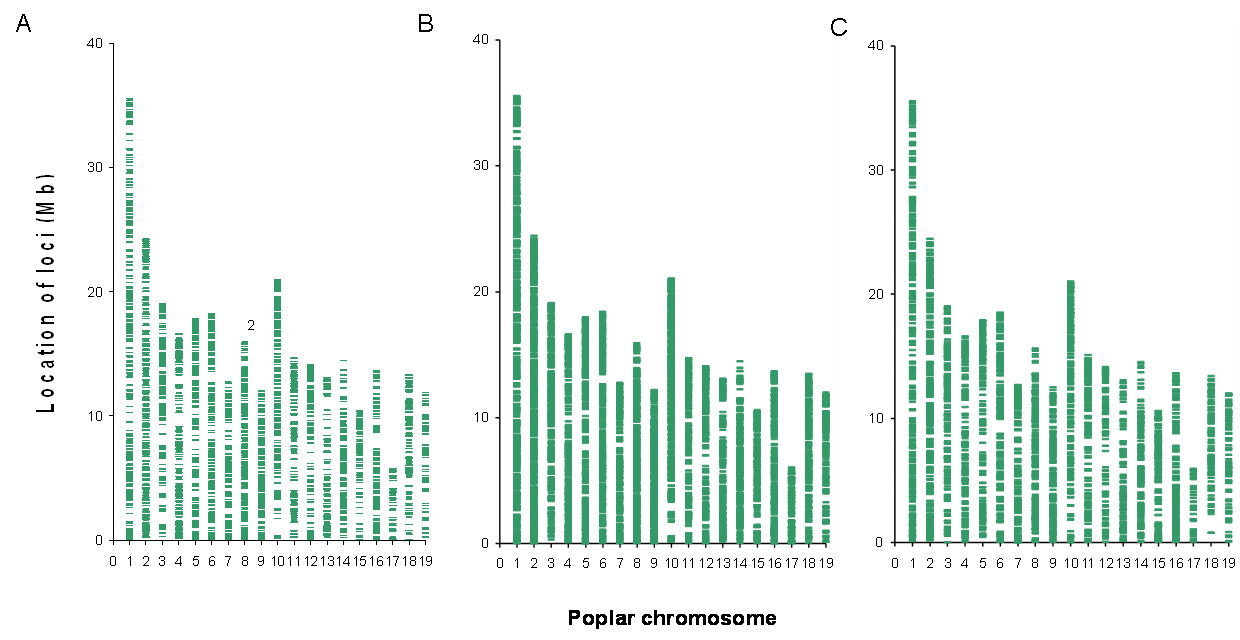

Supplement: Additional file 6 — Location of xylem orthologs on poplar chromosomes. Location of genes homologous to the 527 xylem orthologs in each chromosome of poplar. A: 3,523 stronger homologs based on E ≤ 1e-50; B: 7,436 moderate homologs based on E ≤ 1e-5; C: 3,200 randomly selected loci. [file 1471-2148-10-190-S6.TIFF]
