# Supplementary material for: Comparative genomics reveals conservative evolution of the xylem transcriptome in vascular plants
Source: BMC Evol Biol. 2010 Jun 21;10:190. doi: 10.1186/1471-2148-10-190 (PMC2907377; doi:10.1186/1471-2148-10-190)
Supplement: Additional file 2 — Known protein genes and transcription factors in the xylem transcriptome are relatively more conserved in diverse plants. Known protein genes, unknowns, transcription factors (TFs) and non-TFs of radiata pine, loblolly pine, white spruce and poplar were blasted against gene indices (tblastx) of pine and spruce, and gene models (blastx) of poplar, Arabidopsis, rice, Selaginella and moss. Percentage of hits is presented on the Y-axis at three E-value cut-offs (0, 1e-50 and 1e-5). Different numbers of hits observed with known protein genes and unknowns, as well as with TFs and non-TFs were statistically tested assuming a binomial distribution. Almost all comparisons are statistically significant (P < 0.001) except for a few comparisons between conifers at 1e-5. [file 1471-2148-10-190-S2.PPT]

## Slide 1
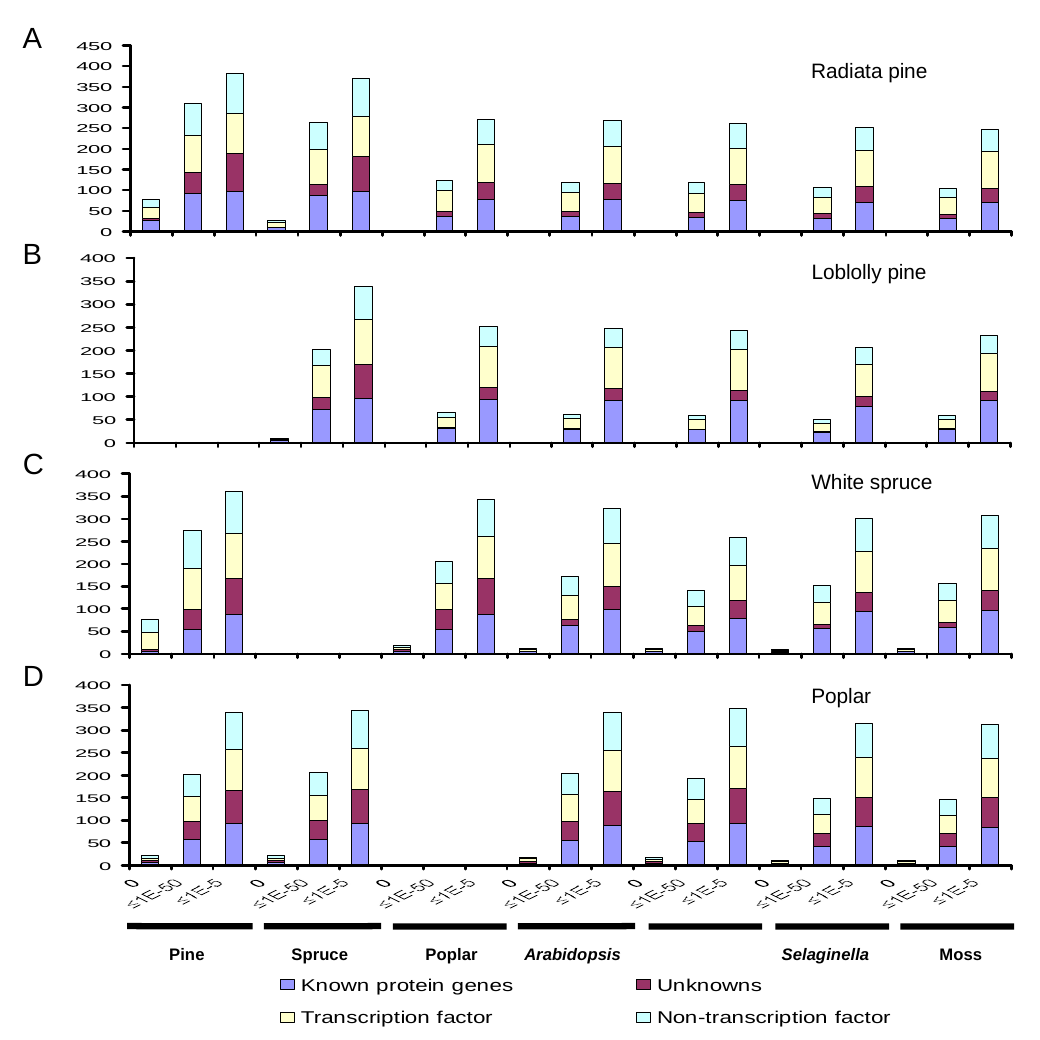

A
 Radiata pine
B
 Loblolly pine
C
White spruce
D
Poplar
 Pine
 Spruce
Poplar
Arabidopsis
 Rice
 Selaginella
 Moss
