# Supplementary material for: Comparative genomics reveals conservative evolution of the xylem transcriptome in vascular plants
Source: BMC Evol Biol. 2010 Jun 21;10:190. doi: 10.1186/1471-2148-10-190 (PMC2907377; doi:10.1186/1471-2148-10-190)
Supplement: Additional file 9 — Public plant genomic resources used in this study. A total of 11 plant species representing different plant groups were selected for this study. Up-to-date public genomic databases were downloaded for comparative genomics analyses. The version and other basic information of these databases are indicated in the table. [file 1471-2148-10-190-S9.DOC]

***Additional file 9 Public plant genomic resources used in this study.*** *ESTs, unigenes, gene indices, gene models and scaffolds from 11 plant species involved in the comparative genomic analysis are summarised in the table.*

| **Species or Genus** | **Gene models**  **(version)** | **Unigenes**  **(version)** | **All ESTs** | **Xylem ESTs ^a^** | **Gene index**  **(version)** | **Xylem gene index ^b^** |
| --- | --- | --- | --- | --- | --- | --- |
| Radiata pine |  | 3304  (PrU1.0) | 5952 | 5952 |  |  |
| Loblolly pine (xylem transcriptome) |  | 20377 ^f^  (Pined20) | 59797 | 59797 |  |  |
| Loblolly pine (entire transcriptome) |  | 18921  (#11) | 292702 | 65266 |  |  |
| *Picea glauca* |  | 17809  (#10) | 88590 | 25389 |  |  |
| *Picea sitchensis* |  | 16755  (#12) | 92411 | 0 |  |  |
| *Populus tremula × Populus tremuloides* |  | 9652  (#15) | 58652 | 17209 |  |  |
| *Populus trichocarpa* | 45555  (v1.1) | 14965  (#9) | 87110 | 11959 |  |  |
| *Eucalyptus grandis* | 14317  (scaffolds) |  |  |  |  |  |
| *Arabidopsis thaliana* | 38963  (TAIR8) | 30383  (#68) | 1041295 | 0 |  |  |
| *Oryza sativa* (Rice) | 67397  (v6.0) | 40973  (#78) | 1163134 |  |  |  |
| *Selaginella moellendorffii* | 34697  (v1.0) |  |  |  |  |  |
| *Physcomitrella patens* (Moss) | 35938  (v1.1) | 18870  (#15) | 376869 |  |  |  |
| *Pinus* ^c^ |  |  | 355326 |  | 61864  (PGI 7.0) | 14527 |
| *Picea* |  |  | 467796 |  | 80494  (SGI 3.0) | 15262 ^d e^ |
| *Populus* |  |  | 411781 |  | 99651  (PplGI 4.0) | 9109  ^d e^ |

^a^**:** ESTs from pure xylem tissues retrieved from the NCBI UniGene database (the mixed xylem tissues, i.e. shoots, are not included).

^b^**:** Tentative consensus (TCs) derived from pure xylem tissues retrieved from the Plant Gene Index database (the mixed xylem tissues i.e. shoots, are not included).

^c^**:** The current version of the pine gene index (PGI 7.0) does not include the radiata pine EST resource.

^d^: All spruce pure xylem gene indices were from white spruce, but poplar pure xylem gene indices were from several poplar species and their hybrids.

^e^: The pure xylem unigenes of loblolly pine and gene indices of white spruce and poplar were reassembled using the same method as in the radiata pine assembly, resulting in 18320, 12489 and 7991 xylem unigenes, respectively.
